# Do Resit Exams Promote Lower Investments of Study Time? Theory and Data from a Laboratory Study

**DOI:** 10.1371/journal.pone.0161708

**Published:** 2016-10-06

**Authors:** Rob Nijenkamp, Mark R. Nieuwenstein, Ritske de Jong, Monicque M. Lorist

**Affiliations:** 1 Department of Experimental Psychology, University of Groningen, Groningen, The Netherlands; 2 Neuroimaging Center Groningen, University Medical Center Groningen, University of Groningen, Groningen, The Netherlands; 3 Research School of Behavioural and Cognitive Neurosciences, University of Groningen, Groningen, The Netherlands; Universita degli Studi di Bologna, ITALY

## Abstract

Although many educational institutions allow students to resit exams, a recently proposed mathematical model suggests that this could lead to a dramatic reduction in study-time investment, especially in rational students. In the current study, we present a modification of this model in which we included some well-justified assumptions about learning and performance on multiple-choice tests, and we tested its predictions in two experiments in which participants were asked to invest fictional study time for a fictional exam. Consistent with our model, the prospect of a resit exam was found to promote lower investments of study time for a first exam and this effect was stronger for participants scoring higher on the cognitive reflection test. We also found that the negative effect of resit exams on study-time investment was attenuated when access to the resit was made uncertain by making it probabilistic or dependent on obtaining a minimal, non-passing grade for the first attempt. Taken together, these results suggest that offering students resit exams may compromise the achievement of learning goals, and they raise the more general implication that second chances promote risky behavior.

## Introduction

Although the most momentous decisions in life may be those we make only once in a lifetime, the majority of our decisions come with the prospect of a second chance. Specifically, there are many situations in which one has to decide on the investment of time and effort to achieve a certain goal (e.g., passing a test) knowing that there will be a second chance to achieve this goal later in time. As a case in point, consider the policies of licensing exams for psychologists and lawyers in the U.S., and those of the many schools that adhere to the learning for mastery system—a system of education that aims for each student to master the to-be-learned materials or skills to a criterion-level of performance [1–3]. Typically, these policies allow an examinee to re-take or “resit” the exam until the exam is passed, thus raising concern about whether it is fair to compare students based on their final grade for the exam ([4]; see also [5–8]).

Aside from concerns about fairness, another potential problem is that resit exams might compromise the achievement of learning objectives because they might lead students to invest less time in studying for an exam. The basis for this concern stems from a recent study by Kooreman [9], who constructed a mathematical model to analyze how resit exams might affect study-time investment for rational students. In constructing this model, Kooreman assumed that such students will strive to maximize utility by optimizing the trade-off between the cost of study-time investment and the benefit of passing the exam. To derive the optimum in this trade-off, Kooreman combined two functions of which the first expressed a concave relationship between study time and passing probability whereas the second expressed a linearly increasing cost of increasing study time. By combining these functions, the optimal study-time investment could be derived as the point at which any additional investment of study time would produce more costs than benefits. In modeling the effect of a resit exam, Kooreman further assumed that rational students will distribute the same amount of time across preparing for a first attempt and resit exam, meaning that the total amount of time invested in preparing for the first attempt and resit exam would not exceed the amount of time invested to pass that exam without a resit. By implication, a resit exam would offer such students the option of investing less time in preparing for the first exam opportunity, knowing that they could still invest additional time so as to be optimally prepared for the resit. As a result, the validity of exam outcomes would be compromised because students who invest less than the optimal amount of time might have a windfall gain in passing that exam. Indeed, by analyzing scenarios with and without a resit exam, Kooreman showed that while the availability of a resit exam increased the overall passing probability by about 8%, the average amount of study time per passed exam was reduced by no less than 39–63% across simulations for different levels of exam difficulty, student ability, and knowledge depreciation between first attempt and resit exam.

Evidently, the results from Kooreman’s [9] analyses are alarming in suggesting that the investment of study time, and hence, the learning of course materials, may be reduced by more than half when students are offered the opportunity to resit an exam. One might question, however, whether this prediction of such a dramatic reduction in study time is indeed accurate. In this regard, it is interesting to consider the results of a study by Grabe [10], who conducted an experimental field study in which resit policy was manipulated for an undergraduate course in educational psychology. Specifically, Grabe examined test scores on a series of three 45-item multiple-choice tests that took place at different moments in the course and that contributed to the score for a final, summative pass-or-fail examination. In doing so, Grabe compared the test scores of three groups of students. The so-called “Conventional” group had only one opportunity for each test. In contrast, the other two groups both had three opportunities for each test (i.e., 2 resit opportunities, spaced at one-week intervals), and these groups differed in that for one group, the score that counted towards the final examination was the best score of all three attempts (the “Best” group), whereas this was the last test score for the other group (the “Last” group). Consistent with the predictions by Kooreman, the results showed that performance on the first attempt was significantly higher for the Conventional (*N* = 88, *M* = 33.6, *SD* = 5.3) than for the Best group (*N* = 96, *M* = 31.3, *SD* = 5.9; *Cohen’s d* = .41), but there was no significant difference between the Conventional and Last group (*N* = 87, *M* = 32.4, *SD* = 6.4). Taken together, these results suggest that the predictions by Kooreman’s model, while in the correct direction, may somewhat overestimate the effect that resit exams have on study-time investment for an initial test. At the same time, however, it is important to note that the study by Grabe should not be interpreted as a conclusive test of Kooreman’s model. This is because the model’s predictions concern the effects of resit exams on study-time investment and passing probability for a criterion-based, pass-or fail-test, whereas Grabe examined the effect of resits on test scores that merely contributed to the score for a final pass-or-fail exam.

### The current study: A modification and empirical test of Kooreman’s model

In the current study, we aimed to conduct a test of the effect of resit exams on study-time investment for a pass-or-fail exam. In doing so, we derived predictions from a modification of Kooreman’s [9] model. We had two important reasons for modifying this model. Firstly, closer scrutiny of the model learned that its parametrization could be improved for the function relating study-time to pass rate. Secondly, we included additional assumptions so as to be able to derive quantitative predictions for a laboratory experiment in which participants had to decide on study-time investment for a fictional exam consisting of 60 multiple-choice items with three answer alternatives. In the following sections, we elaborate on these points as we describe the workings of our model.

### Modeling study-time investment, passing probability and utility for an exam

**Fig 1:**
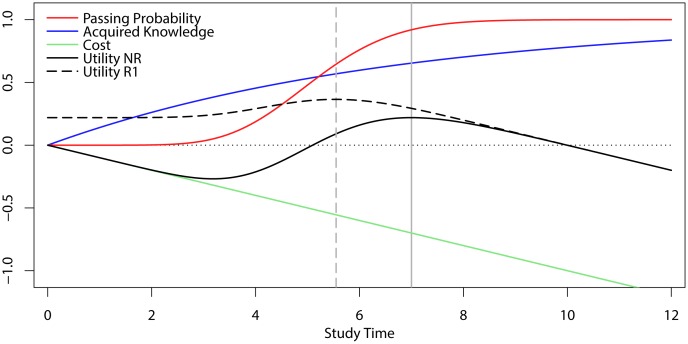
Output of our model relating study time to its costs, the acquired knowledge and associated passing probability for a 60-item multiple choice exam, and the resulting expected utility investing study time for an exam with (R1) or without resit (NR).

The workings of the model we constructed are illustrated in Fig 1, which depicts the model’s assumptions and predictions for the relationship between study-time investment and its costs, acquired knowledge, passing probability, and the resulting net utility, for a multiple-choice exam with or without resit opportunity. A key point of departure from Kooreman’s [9] model was that we began with the assumption that knowledge of course materials will accumulate in an exponential fashion with increasing investments of study time, as suggested by studies of human memory and learning (for a review, see [11]):

$$Proportion\;Acquired\;Knowledge\left(t\right)=p_{know}\left(t\right)=1-e^{\frac{-t}{\beta }},$$ (1)

with *t* denoting study time and *β* denoting the learning rate parameter. This function with *β* = set at 6.6 is shown as the blue line in Fig 1. To model exam performance, we used a multiple-choice exam consisting of 60 items with 3 answer alternatives, and we assumed that for each item, a student would either know the correct answer, with *p*_*know*_(*t*), or else guess randomly. Accordingly, the probability of having a particular number of correct answers, *n*, given study time *t* can be described as the convolution of two density functions, thus summating the number of items predicted to be known and the number of items predicted to be guessed correctly:

h(n,t)=(k*g)(n,t) = Σm=0m=n k(m,N,pknow(t))*g(n−m,N−m,1a), (2)

with functions *k* and *g* denoting binomial probability functions associated with knowing and guessing, respectively, *N* the total number of items, and *a* the number of alternatives per item. Passing probability, given a criterion number of correct answers *n*_*crit*_, is then given by:

$$p_{pass}\left(t\right)=1-H\left(n_{crit}-1,t\right),$$ (3)

with *H* denoting the cumulative probability function associated with *h*. This function is depicted as the red line in Fig 1, for an exam with 60 items that each have 3 answer alternatives, using *n*_*crit*_ = 42 that corresponds to a 56% criterion level of knowledge; a criterion that is a commonly used standard for passing exams at the University of Groningen. Note that this S-shaped function markedly differs from the strictly concave function that was posited for the relationship between study-time investment and passing probability rather arbitrarily by Kooreman [9] (c.f. Figure 1 in [9]). As will be shown below, this difference in the shape of the passing-probability function leads our model to predict more modest effects of resit exams than those suggested by Kooreman’s model.

In modeling the costs of investing study time, we followed Kooreman [9] in assuming that they increase linearly as a function of study time,

$$U_{cost}\left(t\right)=-w*t,$$ (4)

with *w* denoting the cost per unit of study time *t*. This function is shown as the green line in Fig 1. Taken together with the effect of study time on passing probability, this function allows for the calculation of total expected utility as a function of study time, as follows:

$$U_{no\;resit}\left(t\right)=p_{pass}\left(t\right)*U_{pass}-w*t,$$ (5)

with *U*_*pass*_ denoting the utility of passing the exam, set arbitrarily to 1 in our simulations. This utility function is presented as the solid black line in Fig 1, for a case in which *w* = 0.1. As can be seen in this figure, an investment of no study time produces a utility of 0. As the investment of study time increases from 0, utility first decreases because *p*_*pass*_ initially remains close to 0 as the probability of scoring the criterion number of correct items remains low. As the investment of study time increases further, utility starts to increase because passing probability then quickly rises, and eventually peaks at the optimal amount of study time. With even higher levels of study time, however, utility starts to decrease because *p*_*pass*_ increases only marginally beyond the optimum, whereas the cost of study-time investment continues to increase linearly. For future reference, we define *t*_*optimum*_ as the optimal amount of study time in a no-resit condition, *u*_*optimum*_ as the associated maximum utility, and *p*_*optimum*_ as the associated passing probability.

### Modeling the effect of resit exams

We now report how the model described above can be expanded to yield predictions for a case in which students have unconditional access to a resit if they fail to obtain a passing grade for an exam. In doing so, we will use the abbreviations R1 and R2, respectively, to refer to the first exam and resit in a resit condition. To denote exams without resit we will henceforth use the abbreviation NR (no resit).

In expanding the model to a resit condition, we made the simplifying assumption that there is no forgetting of knowledge between R1 and R2. In addition, we followed Kooreman [9] in assuming that if students fail on R1 after investing an amount of study time that is less than *t*_*optimum*_, they will invest an additional amount of study time in preparing for R2 so that the effective total study time equals *t*_*optimum*_ and the probability of passing R2 is *p*_*optimum*_. In case they invested an amount of study time equal to or more than *t*_*optimum*_ for R1, we assumed that they will not invest any additional study time for R2. Thus,

$$t_{R2}=max\left(t_{optimum}-t_{R1},0\right),$$ (6)

$$p_{pass\;R2}=max\left(p_{optimum},p_{pass}\left(t_{R1}\right)\right)$$ (7)

Under these assumptions the expected utility for study-time investments for R1 can be derived and computed using the following utility function:

$$U\left(t_{R1}\right)=\left(p_{pass}\left(t_{R1}\right)+\left(1-p_{pass}\left(t_{R1}\right)\right)*p_{pass\;R2}\right)*U_{pass}-w*\left(t_{R1}+\left(1-p_{pass}\left(t_{R1}\right)\right)*t_{R2}\right)$$ (8)

As can be seen by inspecting the resulting utility function in Fig 1, the expected utility for R1 remains fixed at the level of *u*_*optimum*_ for investments of up to about 3 units of study time. This reflects the fact that for such investments, the probability of passing R1 is close to zero, in which case students are assumed to invest an additional amount of study time to achieve *t*_*optimum*_ and *p*_*optimum*_ for R2. As study-time investment increases beyond this point, expected utility starts to increase and reaches a maximum that is distinctly higher than *u*_*optimum*_, at a distinctly lower amount of study time than *t*_*optimum*_ in the condition without resit, after which it drops off again for the same reason stated before for the no-resit condition.

### The resit effect

What can be seen from comparing the utility functions for the first attempt in the resit condition (R1) and the single attempt in the no-resit condition (NR) in Fig 1 is that the maximum expected utility for an exam with resit is reached at a level of study-time investment that is lower than for an exam without resit. Specifically, the model suggests that the optimal investment may drop from 7 to 5.5 units, yielding a decrease of 20% of study time and a corresponding 12% decrease in acquired knowledge. Thus, in line with Kooreman’s [9] model, our model also predicts that resit exams will promote lower investment of study time. Importantly, however, the predicted magnitude of this effect is less dramatic than that predicted by Kooreman’s model [9]. This difference between the models can be traced completely to the difference between the shapes of the passing probability function, *p*_*pass*_*(t)*, with the S-shaped function of the present model stemming directly from the combination of an exponential learning curve and the statistical properties of multiple-choice tests.

## Experiment 1: A Test of the Model’s Predictions

**Fig 2:**
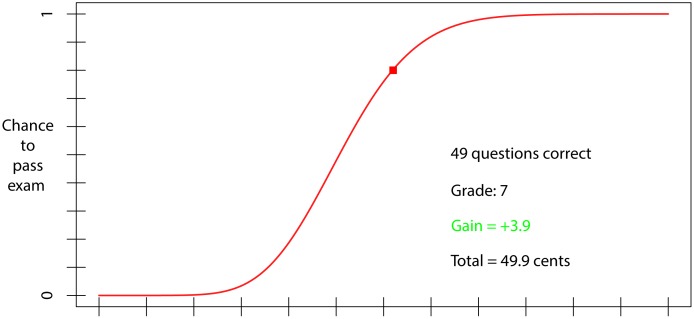
A plot showing the relationship between study-time investment (x-axis) and the probability of passing a simulated exam (y-axis) that was used as the stimulus across the two experiments. Also depicted is the feedback that was presented after study time was invested.

To test the predictions of our model, we designed an investment task in which participants had to invest fictional study time to pass a fictional exam, under conditions with and without a resit. Importantly, on each trial participants were shown the same passing-probability function (see Fig 2; note that this is the same function as shown in Fig 1 and discussed in the text) and they were informed about the linear time-cost function. They then indicated the amount of study time that they wished to invest. Thus it was not our aim to test the model’s assumptions that underlie the predicted passing-probability function or the assumed linear time costs. Rather, each participant was confronted with exactly the same optimization problem and provided with all the necessary information to arrive at optimal investments. In our perspective, this is a useful and perhaps even necessary intermediate step toward attempts to investigate possible resit effects in more naturalistic and realistic settings. If participants were to show resit effects in these highly controlled conditions, this would be an interesting, novel, and useful result that should motivate subsequent efforts to investigate such effects in more realistic settings. If, on the other hand, resit effects would fail to show up in these controlled conditions, the possibility that they would show up in more natural conditions would seem extremely remote, as such conditions typically come with many complicating factors, such as individual differences in prior knowledge and experience, learning capabilities, and subjective costs of study-time investments, the forgetting of knowledge over time, imperfectly designed exams, far more varied and less precise subjective estimates of passing-probability functions, that would surely compromise the reliable and consistent expression of such effect.

At each trial, the invested study time was translated into *p*_*know*_ and, using the simple binary model for a 60 item, 3-alternative multiple-choice exam, this value was used to generate a probabilistic exam score consisting of the number of correct answers (0–60) and associated grade (0–10; in steps of one point). If this grade was a 6 or higher (rounded up from 5.6, the minimal knowledge level required for passing the exam) the participant passed the exam. In the resit condition, participants had 2 opportunities to pass the exam. To increase the likelihood that participants would take this investment task seriously and try to optimize expected utility, we used monetary pay-offs such that each unit of study time cost 1 cent, whereas passing the exam produced a monetary reward of 10 cents. Lastly, to determine whether the effect of resit exams is associated with rationality, we had participants complete the Cognitive Reflection Test (CRT; [12])–a test that is known to be associated with performance on several indices of rationality in judgment and decision making [13].

### Predictions examined

To the extent that participants act rationally in seeking to maximize expected utility, our model yields several predictions for study-time investment and passing probability on exams done with and without resit. Specifically, the model predicts that time investment would be equal for NR and for R2 (c.f. Eq 6), whereas it should be about 20% lower for R1. Secondly, as can be derived from Eq 9, the model predicts that the overall passing probability will be higher in the resit condition than in the no-resit condition. This is because the chance of passing R2 would be expected to be equal to the chance of passing NR, while there would be a windfall gain for the resit condition stemming from the probability that students could also pass R1. Importantly, since the amount of time invested on such passed R1s is expected to be lower than that invested time for passed NRs, it furthermore follows that the average amount of study time invested per passed exam would be lower overall in the resit condition. Lastly, since the model takes a rational calculation of expected utility as its starting point, one would also expect that the effect of resit exams on study-time investment would be stronger for participants who score higher on the CRT [12].

### Method

#### Participants

Fifty first-year psychology students (21 male) from the University of Groningen participated in the experiment in return for course credit. Their mean age was 20.6 years (*SD* = 2.27). Written informed consent was obtained from all participants prior to the experiment. Additionally, ethical approval was obtained from the Ethical Committee Psychology at the University of Groningen before the start of data collection.

#### Materials

The study-time investment task was programmed in MATLAB and run on computers situated in a room with 5 computer set-ups, which were enclosed by paperboard walls. The stimulus for the task consisted of a graph which depicted the relationship between study time, defined in terms of 12 arbitrary units along the x-axis, and the probability of passing the 60-item multiple choice exam along the y-axis (Fig 2)—this function remained the same throughout the experiment. The graph included a cursor that could be moved along the curve by the participant. Prior to starting the investment task, participants completed the CRT [12].

#### Design and procedure

The study-time investment task required participants to indicate their choice of study-time investment for passing an exam. They were informed that they would pass the exam if their grade was a 6 or higher. To select a desired amount of study time, participants could move a cursor along the curve in the graph, and they had to click the mouse to select the amount of study time they wanted to invest.

The availability of a resit was manipulated within-subjects in a blocked design, such that each participant completed 6 blocks of 60 trials, of which 3 blocks included a resit option whereas the other blocks did not. The resit and no-resit conditions were presented in alternation and half the participants started with the resit condition whereas the other half started with a no-resit condition. At the start of the first two blocks, participants completed 5 practice trials. Prior to starting the task, participants were informed about the nature of the exam, the required number of correct answers to pass the exam, and the nature of the relationship between study-time investment and the probability of passing the exam. In addition, participants were informed that they could earn real money such that they would obtain a reward of 10 cents if they passed the exam, with the cost of study time being 1 cent per unit invested. A failure to pass the exam produced no reward. After deciding on their investment, participants were informed of the number of correct answers and the resulting grade and earnings, and they also received information about their total net earnings up to that point in the task.

In the resit condition, participants moved on to the next trial if a passing grade was obtained on R1. If R1 yielded a failing grade, they moved on to R2 after the result of R1 had been presented for 2.5 seconds. For R2, the choice of study-time investment was restricted to an amount equal or higher than the study time invested for R1. This was made clear by presenting the cursor on the study time invested for R1 and by coloring the curve green above this point, thus highlighting which additional amount of time could be invested for R2.

#### Data analysis

In presenting the results for both experiments reported here, listed study times for R2 represent total effective study times (study time used to prepare for R1 + any additional study time used to prepare for R2). To test our predictions, we used the JASP software package [14] to compute Bayes factors as a means to assess the extent to which the results provided evidence in favor or against our predictions [15]. Specifically, we used Bayesian paired samples t-tests to compare NR, R1, and R2 in terms of study-time investment, passing probability, and study-time per passed exam and we used one-sample *t*-tests to compare study time and passing probability to the point-estimates derived from our model. Lastly, we used a Bayesian correlation to assess the evidence for a positive correlation between participants’ CRT scores and the resit effect, defined in terms of the difference in study-time investment for NR and R1. When we predicted a difference in a specific direction (e.g., study time R1 < R2), we used a one-sided test to contrast the evidence for this predicted effect against a null effect. For predicted null effects (e.g., study-time investment on R1 was predicted to be equal to the optimal amount of study time derived from our model), we used a two-sided test thus specifying inequality as the alternative to the predicted equality of study-time investment. Thus, all Bayes factors were computed in such a way that values greater than 1 would indicate evidence in favor of the predicted effect, whereas values between 0 and 1 would indicate evidence against the predicted effect. In reporting the results of these Bayes factors analyses we adhered to Jeffreys [16] in classifying *BFs* > 3 or < .33 as “strong” evidence, whereas *BFs* > 100 or < .01 were classified as “decisive” evidence.

#### Outlier exclusion

Investments of less than 2 or more than 10 study-time units were classified as outliers and excluded from further analysis. This entailed a loss of 0.4% of the trials. A comparison of the results with and without these outliers showed that outlier exclusion did not affect the results.

### Results

Table 1 lists the results of Experiment 1 while Table 2 lists our predictions and the results of the Bayes factors analyses we did to test these predictions. As can be seen by inspecting these data, the results produced decisive evidence in favor of our main prediction that participants would invest less study time in preparing for an exam when a resit was available. Specifically, the results confirmed that participants invested less time in preparing for R1 than for NR and that they invested less time for R1 than for R2. Concomitantly, passing probability was lower for R1 than for NR, and lower for R1 than for R2. Furthermore, the results also make clear that a substantial percentage of R1s were passed in spite of lower investments of study time. As a result, the overall probability for passing an exam was higher in the resit condition, but the average amount of study time invested per passed exam was lower for this condition.

**Table 1:** Results Experiment 1.

Exam	Study Time		% Passed		Study Time per Passed Exam
	Mean (*SD*)	Model	Mean (*SD*)	Model	Mean (*SD*)
**NR**	6.2 (.5)	7.0	77.6 (9.5)	92.0	6.3 (.5)
**R1**	5.2 (.7)	5.5	53.7 (17.8)	64.5	5.4 (.7)
**R2**	6.5 (.7)	7.0	78.6 (8.6)	92.0	6.4 (.7)
**R1&R2**	5.9 (.7)	7.0	89.2 (6.2)	96.7	5.8 (.5)

Model predictions and data for study-time investment and percentage passed are shown together with the average amount of study time per passed exam. NR = exam in no-resit condition, R1 = first exam in resit condition, R2 = resit exam in resit condition, R1&R2: results averaged across the first attempt and resit exam in the resit condition. *M* = mean, *SD* = standard deviation.

**Table 2:** Results of Bayes factors analyses for Experiment 1.

Outcome Measure	Prediction	Bayes Factor Prediction vs. Alternative
**Study Time**	NR > R1	3.7 x 10^13^
	NR = R2	5.8 x 10^−4^
	R1 < R2	7.3 x 10^18^
	NR > R1&R2	45857
	NR = Model NR	8.7 x 10^−12^
	R1 = Model R1	0.21
	R2 = Model R2	0.001
	[R1&R2] = Model	4.9 x 10^−14^
**% Passed**	NR > R1	3.8 x 10^11^
	NR = R2	4.5
	R1 < R2	2.8 x 10^13^
	NR < R1&R2	7.6 x 10^13^
	NR = Model NR	3.2 x 10^−12^
	R1 = Model R1	0.003
	R2 = Model R2	1.0 x 10^−12^
	[R1&R2] = Model	2.9 x 10^−9^
**Study Time per Passed Exam**	NR > R1	6.5 x 10^13^
	NR = R2	1.0
	NR < R1&R2	3.0 x 10^14^
	R1 < R2	1.2 x 10^16^

Outcome measure denotes the outcome measure examined, prediction specifies the effect by the model. All Bayes factors express the ratio of evidence for the predicted effect against the alternative. Thus, values greater than 1 signify evidence in favor of the prediction, whereas values between 1–0 express evidence for the alternative, and a value of 1 signifies no evidence in either direction. *BFs* > 3 or < .33 are considered as strong evidence whereas *BFs* > 100 or < .01 are considered as decisive evidence [16].

Aside from corroborating the predicted differences in study-time investment and passing probability, the results also produced a number of outcomes that were inconsistent with our predictions. Specifically, while we predicted that participants would invest an equal amount of study time for R2 and NR, the results yielded decisive evidence for a difference in study-time investment, such that more time was invested for R2 than for NR. Importantly, however, the analysis of passing probability showed that this difference in study-time investment did not result in a difference in passing probability, as the analysis of passing probability produced strong evidence for equal passing probabilities for R2 and NR. A second regard in which the results deviated from predictions was that the participants did not invest the predicted optimal amount of study time for each type of exam, such that participants invested less than the predicted amount of study time for all three types of exams, and for the resit condition overall. As a result, passing probability was lower than predicted by the model for all exams as well.

Lastly, we examined whether there was a positive correlation between the resit effect, defined in terms of the difference in study-time investment for NR and R1, and the participants’ CRT scores. Examination of performance on the CRT showed that on average, participants answered 1.26 of the three questions correctly (*SD* = 1.03), with 14, 16, 13, and 7 participants having 0, 1, 2, and 3 correct answers, respectively. A Bayesian test of whether CRT scores were positively correlated with the resit effect yielded a correlation of *r* = .34 and a Bayes factor of 6.1, indicating that the data produced strong evidence in favor of the existence of a positive relation between CRT scores and the effect of resit exams on study-time investment.

### Discussion

Taken together, the results of Experiment 1 can be said to largely conform to our main predictions, as participants were found to invest less time in preparing for an exam when a resit was available, and this effect was furthermore found to be stronger for participants with high scores on the CRT [12]. Importantly, however, there are also two regards in which the data did not conform as closely to our model’s assumptions and predictions. Firstly, the results showed that study-time investment for the different types of exams was lower than the optima derived from our model, indicating that participants did not succeed in maximizing utility because they invested less than the optimal amount of study time for passing the exams. Secondly, we found that participants invested more time for resit exams (R2s) than for exams without resits (NRs) whereas our model assumes that an equal amount of study time would be invested for these two kinds of exams. Importantly, however, the difference in study-time investment for NR and R2 did not yield a difference in passing probability. The reason for this can be seen in Fig 1, which shows that the functions relating passing probability and expected utility to study time are relatively flat at the optimal amount of study time predicted by our model. By implication, minor deviations from the optimum amount of study time were relatively inconsequential for passing probability and utility, thus allowing participants to attain close to the maximally attainable utility even when investing somewhat less than the optimal amount of study time.

## Experiment 2

The results of Experiment 1 make clear that in investing fictional study time for a fictional exam, the availability of a resit exam causes students to invest less time in preparing for an exam. As a result, a considerable percentage of students may pass the exam without having gained mastery of the required amount of course materials. In light of this concern, an important question is how the effect of resit exams on study-time investment might be mitigated. In addressing this matter, it is important to note that access to resit exams is typically unconditional, meaning that there are no detrimental consequences to not passing an exam, as one could always still invest more time to be optimally prepared for the resit. But what if access to resit exams would no longer be guaranteed, for instance because one needs a certain minimal grade, or because access to the resit is uncertain because it is probabilistic? Arguably, such restrictions on access to resit exams would reduce the risk that students do not study hard enough to acquire sufficient knowledge to pass the exam, as this would now entail a risk of not getting access to the resit exam. Accordingly, imposing restrictions on access to a resit exam could mitigate the resit effect by getting students to invest more time in preparing for the first exam opportunity.

In Experiment 2, we examined and tested the predictions of our model for how study-time investment would be affected in case access to a resit exam is probabilistic or conditional on obtaining a minimal non-passing grade. Specifically, we replicated the unconditional resit (UR) and no-resit (NR) conditions of Experiment 1, and we included two new conditions in which access to the resit was conditional on obtaining a minimal grade of 4 (i.e., the grade-based resit condition, GR) or on chance, such that there was only a 50% probability of gaining access to the resit in case a non-passing grade was obtained on R1 (i.e., the probabilistic resit condition, PR). For the latter condition, we derived model predictions using formula 9, whereas the predictions for the former condition were derived from formula 10:

$$U_{PR}\left(t_{R1}\right)=\left(p_{pass}\left(t_{R1}\right)+\left(1-p_{pass}\left(t_{R1}\right)\right)*p_{access\;R2}*p_{pass\;R2}\right)*U_{pass}-w*\left(t_{R1}+\left(1-p_{pass}\left(t_{R1}\right)\right)*p_{access\;R2}*t_{R2}\right),$$ (9)

$$U_{GR}\left(t_{R1}\right)=\left(p_{pass}\left(t_{R1}\right)+\left(p_{access\;R2}\left(t_{R1}\right)-p_{pass}\left(t_{R1}\right)\right)*p_{pass\;R2}\right)*U_{pass}-w*\left(t_{R1}+(p_{access\;R2}\left(t_{R1}\right)-p_{pass}\left(t_{R1}\right)\right)*t_{R2},$$ (10)

where *p*_*access R2*_*(t*_*R1*_*)* is analogous to the derivation of *p*_*pass*_*(t)* in Eq 3:

$$p_{access\;R2}\left(t_{R1}\right)=1-H\left(n_{crit,\;accessR2}-1,\;t_{R1}\right),$$ (11)

with *n*_*crit*, *access R2*_ denoting the minimum number of correct answers required to score a grade of 4 or higher (*n*_*crit*, *access R2*_ = 34; the critical number of 34 correct answers corresponds to a knowledge level of at least 35%, which is required to score at least a 4 on the exam).

**Fig 3:**
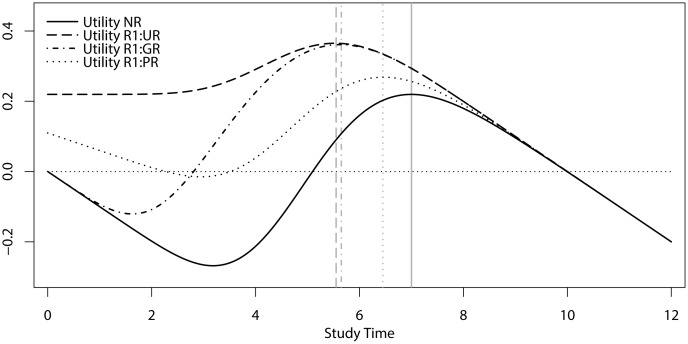
Model output for the utility associated with investing study time for an exam without a resit (NR) or with a resit for which access is unconditional (UR), based on obtaining a minimum non-passing grade of 4 (GR), or probabilistic with a 50% chance (PR).

*Predictions*. As can be seen in Fig 3, our model predicts that the optimal amount of time investment for the conditions with restricted access to the resit lies between the optima of the resit condition with unrestricted access and the no-resit condition. Specifically, as can be seen in this figure, our model predicts that the resit effect will be weakest in the PR condition, stronger in the GR condition, and strongest in the UR condition.

### Method

#### Participants

The participants in Experiment 2 were 48 students (14 male, *M*_age_ = 19.8, *SD*_age_ = 3.23) from the University of Groningen who participated in return for course credit. Written informed consent was obtained prior to participation in the experiment. Additionally, ethical approval was obtained from the Ethical Committee Psychology at the University of Groningen before the start of data collection.

#### Design and procedure

The four conditions (i.e., NR, UR, GR, and PR) were implemented as a within-subjects factor in a blocked design. That is, for each condition, participants completed one block of 60 trials that was preceded by 5 practice trials. The reason for not including 3 blocks per condition, as we did in Experiment 1, was that further analyses of the results of Experiment 1 showed that the difference between the resit and no-resit conditions was already evident in comparing the first block of each condition. Each possible order of the four conditions was used twice across the 48 participants. The procedure of Experiment 2 was the same as in Experiment 1, with exception of the two added conditions in which access to the resit exam was either probabilistic or contingent on obtaining a grade of 4 or more points. Another change in the procedure was that Experiment 2 did not make use of monetary incentives, meaning that participants did not earn or loose actual money.

#### Data analysis

In analyzing the results of Experiment 2, we followed the same procedures as those used for data analysis in Experiment 1. The analyses reported below aimed to test our predictions that each of the three resit conditions would yield a resit effect, with the magnitude of this effect being strongest for UR, weaker for GR, and weaker still for PR. In addition, we examined if the results of Experiment 2 replicated Experiment 1’s unpredicted result of higher investments of study time for UR2 than for NR.

#### Outlier exclusion

Investments of less than 2 and more than 10 units of study time were classified as outliers and excluded from further analysis. As a result, a total 0.5% of all trials were excluded. A comparison of results with and without outliers showed that the exclusion had no significant impact on the results. In addition, we had to exclude the data from two participants, because they did not yield data for one or more resit conditions because of passing all exams on the first attempt. Accordingly, the results reported below stemmed from 46 participants.

### Results

The results of Experiment 2 are listed in Tables 3 and 4 presents the outcomes of the Bayes factors analyses. The results conformed fully to our predictions. Specifically, they showed that equal amounts of study time were invested for NR and UR2, and they showed that, compared to NR, study-time investment and passing probability were lower for the first exam attempt in each of the three resit conditions. In addition, the results showed that the resit effect was strongest in the UR condition, weaker in the GR condition and weakest in the PR condition. As in Experiment 1, actual study-time investments generally were somewhat lower than the optimal values predicted by our model.

**Table 3:** Results Experiment 2.

Exam	Study Time		% Passed		Study Time per Passed Exam
	Mean (*SD*)	Model	Mean (*SD*)	Model	Mean (*SD*)
**NR**	6.4 (.6)	7.0	81.2 (10.9)	92.0	6.5 (.6)
**UR1**	5.4 (.8)	5.5	57.5 (20.8)	64.5	5.6 (.8)
**UR2**	6.5 (.8)	7.0	80.6 (12.9)	92.0	6.6 (.9)
**UR1&UR2**	6.0 (.7)		90.5 (9.1)		6.0 (.5)
**GR1**	5.7 (.8)	5.7	63.2 (18.6)	67.3	5.8 (.8)
**GR2**	6.7 (1.0)	7.0	81.5 (12.2)	92.0	6.8 (.9)
**GR1&GR2**	6.2 (.8)		91.1 (8.0)		6.1 (.6)
**PR1**	5.9 (.6)	6.5	70.1 (15.5)	84.8	6.0 (.6)
**PR2**	6.7 (.8)	7.0	82.9 (16.5)	92.0	6.8 (.8)
**PR1&PR2**	6.3 (.7)		81.9 (10.7)		6.1 (.5)

Model predictions and data for study-time investment and percentage passed are shown together with the average amount of study time per passed exam. NR = exam in no-resit condition, UR1 and UR2 = the first and resit exam in unconditional resit condition, UR1&UR2 denotes overall results for the unconditional resit condition, meaning the results for both R1 and for UR2 on trials in which UR1 was failed. Same terminology applies for GR = grade restricted resit condition, and PR = probability restricted resit condition. *M* = mean, *SD* = standard deviation.

**Table 4:** Results of Bayes factors analyses for Experiment 2.

Outcome Measure	Prediction	Bayes Factor Prediction vs. Alternative
**Study Time**	NR = UR2	4.3
	NR > UR1	9.7 x 10^10^
	NR > PR1	95196
	NR > GR1	1.4 x 10^8^
	RE_UR > RE_GR	16.6
	RE_GR > RE_PR	3.3
**% Passed**	NR = UR2	5.9
	NR > UR1	7.8 x 10^8^
	NR > PR1	1.7 x 10^6^
	NR > GR1	36421
	GR1 > UR1	5.4
	PR1 > GR1	24.5

Outcome measure denotes the outcome measure examined, prediction specifies the effect by the model. All Bayes factors express the ratio of evidence for the predicted effect against the alternative. Thus, values greater than 1 signify evidence in favor of the prediction, whereas values between 1–0 express evidence for the alternative, and a value of 1 signifies no evidence in either direction. *BFs* > 3 or < .33 are considered as strong evidence whereas *BFs* > 100 or < .01 are considered as decisive evidence [16]. RE_UR denotes the magnitude of the resit effect on study-time investment for the unconditional resit condition. Same terminology applies for the other two resit conditions.

As in Experiment 1, we aimed to use the results of Experiment 2 to test the correlation between the magnitude of the resit effect and performance on the CRT. Unfortunately, however, the participants’ CRT scores in Experiment 2 did not allow for a proper assessment of this correlation, as only 2 participants had 3 questions correct, whereas the number of participants with 0, 1, 2 correct answers was 8, 28, and 8. Accordingly, we decided to combine the data across Experiments 1 and 2 to assess the correlation between the resit effect and CRT, for the resit effect for the condition with unconditional access to a resit. A Bayesian correlation analysis showed that these data reaffirmed our finding in Experiment 1, showing a positive correlation of *r* = .25 with a Bayes factor of 4.6, thus indicating that the results offered strong support for a positive correlation between the resit effect and CRT.

### Discussion

Consistent with the predictions of our model, the results of Experiment 2 show that restricting access to resit exams might be an effective method to mitigate the reduction of study-time investment in preparing for a first attempt at passing an exam. Specifically, the results show that participants invested more fictional study time in preparing for a fictional exam when access to the resit was probabilistic or conditional on obtaining a minimum grade of 4, with the former being most effective in countering the effect of resit exams on study-time investment. In addition, an analysis combining the results for Experiments 1 and 2 replicated the finding of a positive correlation between the magnitude of the resit effect and performance on the CRT [12]. Lastly, it is worth noting that the results of Experiment 2 differed from those of Experiment 1, in that they provided evidence that participants invested an equal amount of study time for unconditional resit exams and exams without resit, an effect that was assumed by our model, but not found in Experiment 1. In considering an explanation for this difference, it is important to note that the difference in study-time investment found in Experiment 1 was inconsequential for the passing probability, and that furthermore, the results of Experiment 2 showed a numerical difference in the same direction as that found in Experiment 1. Hence, it appears to be the case that participants may invest slightly more study-time for a resit exam than for an exam without resit, perhaps reflecting a tendency to overcompensate on the resit after a failure to pass the first attempt at an exam—a possibility discussed further in detail below in the General Discussion.

## General Discussion

In the current study, we followed up on the work by Kooreman [9] and Grabe [10] by investigating how resit exams might influence the investment of study time and, concomitantly, the probability of passing an exam. To this end, we modified Kooreman’s model of resit exams by including some well-justified assumptions about learning and performance on multiple-choice tests, and we conducted two experiments to test the predictions of the model. In these experiments, participants were asked to invest study time for a fictional pass-or-fail exam that either did or did not include a resit opportunity. Consistent with our model’s predictions, the results of Experiment 1 showed that resit exams promoted lower investments of study time in preparing for an exam, and this effect was stronger for participants scoring high on a test of rationality, the CRT [12]. Importantly, since a substantial percentage of the exams with lower investments were still passed, the average amount of study time per passed exam was lower when a resit exam was available. To determine how this unwanted effect of resit exams might be countered, we modeled and tested the effect of restricting access to resit exams in Experiment 2. Consistent with our predictions, the results of this experiment showed that the resit effect was attenuated when access to the resit was uncertain because it was probabilistic or based on obtaining a minimal non-passing grade.

### Rationality or reactive decision making?

In interpreting the current results, an interesting question is whether the resit effect indeed stems from a rational attempt at maximizing utility, as assumed by our model (see also [9]), or whether other factors might also contribute to this effect. In addressing this question, it is of relevance to note that there are different lines of research that show that participants tend to react to losses and gains, such that a loss is more likely to be followed by a safe choice whereas a gain is more likely to be followed by a riskier choice. For instance, studies on response caution show that when participants make an error in a speeded judgment task they will typically respond more slowly on the next trial, thus suggesting that there is an increase in decision threshold to safeguard against another mistake on the next trial (for a recent review of this literature, see [17]; see also [18]). Likewise, in a paradigm more closely related to the current resit paradigm, Kareev, Avrahami, and Fiedler ([19]; see also [20]) showed that a financial decision yielding a gain is more likely to be followed by a risky decision than a decision that yielded a loss.

The relevance of these findings to interpreting the current results lies in the fact that in our study, the overall pass rate was significantly higher in the conditions involving resit exams, meaning that these conditions included more trials in which the participant’s investment decision on a previous exam yielded a gain. Thus, one could argue that perhaps the lower investment of study time in the resit condition was driven by a reaction to the gain of passing a previous exam, and not by a rational expected utility analysis. Importantly, however, further examination of the current dataset makes clear that this hypothesis cannot account for the resit effect we found. To be precise, we examined this hypothesis in a post-hoc analysis which included the results for the no-resit and unconditional resit conditions of Experiments 1 and 2, and which compared for each condition the trials in which the previous exam was passed or failed (i.e., NR_PreviousPassed_−NR_PreviousFailed_ and R1_PreviouPassed_−R1_PreviousFailed_). The results of this analysis showed that passing on the previous trial, as compared to failing, did not lower study-time investment on the current trial, thus arguing against the idea that the lower investment of study time for a first exam in the resit condition was driven by a reaction to the outcome of the previous trial. Specifically, when considering only those trials in which the previous trial yielded a non-passing grade on the exam, the average amount of study time invested for NR and R1 were 6.1 (*SD* = 0.5) and 5.1 (*SD* = 0.6), respectively. For trials preceded by a trial yielding a passed exam, the amounts of study time invested for NR and R1 were 6.2 (*SD* = 0.5) and 5.2 (*SD* = 0.6), respectively. Bayes factors analyses comparing the effects of passing (Previous Passed—Previous Failed) for the two conditions showed no evidence for lower study-time investment after passing, with Bayes factors of 17.1 and 31.3 in support of the null hypothesis for NR and R1, respectively.

### Why were investments suboptimal?

Another aspect of the current study that deserves further scrutiny lies in the fact that participants invested less than the predicted optimal amounts of study time for all exam types considered. At present, we can conceive of two reasons why participants invested less than the optimal amounts of study time. The first is that participants may have subjectively overestimated their likelihood of passing the exam when the actual passing probability they chose by means of their investment was relatively low. This would be consistent with the certainty effect epitomized by prospect theory’s pi-function [21], which denotes the fact that people generally overestimate the likelihood of low-probability events. Alternatively, the finding of lower-than-optimal investments could be explained by assuming that participants behaved risky in an attempt to “beat the odds” in passing the fictional exam. While such a risky strategy might be less likely in case of preparing for a real exam, it is important to bear in mind that such real exams typically do come with unconditional access to resit exams and this surely will not discourage a risk-seeking student from following this strategy.

### Resits and goal pursuit

In demonstrating that resit exams promote lower investment of study time for a fictional exam, the current findings can be said to resonate well with two recent findings from studies on goal pursuit. Specifically, these studies suggest that people are less committed to a particular means to achieving a goal when more such means are available [22,23] and they suggest that participants are less likely to achieve a goal (e.g., achieving high performance on a sentence unscrambling task so as to receive a free snack) if they have first thought of a back-up plan for achieving that goal (i.e., think of another way in which you can obtain free food on campus; [24]; see also [25]). Interpreted in view of these findings, one could argue that in the current study, the prospect of a resit exam led participants to be less committed to passing the exam on their first attempt, as the resit exam offered them an alternative means in the form of a back-up plan. An important consideration that derives from the current work is that this lack of commitment is rational, because it entails an attempt to maximize utility by trying to achieve one’s goal with the least amount of effort.

### Resits and repeated gambles

Another line of research that is of interest to discuss in the context of the present findings can be found in studies investigating the repeated gambles effect—the finding that participants are more likely to choose a risky gamble or to accept a risky bet when this gamble or bet is played repeatedly, as opposed to only once ([26]; see also, [27–29]). The similarity between this effect and the resit effect lies in the fact that both show that participants are inclined to take or accept more risk when a choice or a bet is repeated. The important difference, however, lies in the fact that in the repeated-gambles paradigm, the participants are asked to commit themselves to the same choice for each opportunity, such that they have to choose the same option for each of a series of repeated gambles, or decide whether they are willing to play the same bet several times in a row. With this design, the outcome can be said to reflect the participants’ ability to oversee the long-run consequences of following a particular strategy, that is, to gauge whether the risky gamble or bet might be profitable in the long run. In contrast, the resit effect described here pertains to how the prospect of a second chance influences a first choice. In this case, the first choice is not based on explicit calculation or intuition about the expected outcome of repeating that choice, but on the mere fact that there will be a second chance to do things differently in case the first decision turns out badly, a back-up plan so to speak ([24]; see also [25]). Thus, the current findings can be said to supplement the repeated-gambles effect in demonstrating another important exception to the well-established notion that people are generally risk averse in decisions involving gains.

### Limitations and extensions of the model

Although the current study provides a first set of findings indicative of the potential effects of resit exams on study-time investment, the current work did not address the full scope of this issue. Specifically, the present research focused on the core assumption of our model, namely that the way in which students allocate study time under different examination rules can be aptly described as rational, with students striving to maximize expected utility by seeking an optimal trade-off between the costs of study-time investment and the (probabilistic) benefits of passing the exam. That the results were in good accordance with the predictions of the model is an important outcome, but it obviously represents only a first step towards a more complete understanding of whether and how different types of examination rules and policies may influence study behavior in higher education. To generate clear and unambiguous predictions for the present study, the model was effectively stripped of much of its relevant scope and functionality. For instance, in its full form the model incorporates mechanisms to assess and predict effects on study-time investment for a wide range of examination rules and various psychological factors such as forgetting of acquired knowledge from R1 to R2, students’ ambitions to obtain high grades rather than seeking to pass exams with minimal effort and minimal grades, and temporal discounting of future study-time costs and benefits for R2 as compared to R1. In addition, the model can also be adjusted in terms of its assumptions about the costs of study-time investment, which in reality may take on more complicated forms than the linear relationship we incorporated in the current version of the model. Some of these effects will be briefly discussed, in order to provide a broader perspective on the results of the present study.

To start, it is of relevance to note that our choice for the value of the learning rate parameter (*β* = 6.6) merely served to define the dynamic temporal range of the model. With the utility of passing the exam fixed at 1, the time-cost parameter w represents the cost per unit of study time as a fixed proportion of that utility. The choice of w = 0.1 in the present work was motivated solely by the fact that it yielded a tradeoff with fairly distinct optima for the expected-utility functions. Much larger values for w (which, for instance, might represent a scenario in which time is at a premium due to other time-consuming obligations) result in non-positive expected utilities for any nonzero time investment, making t = 0 (passing up on preparing for and taking the exam) the optimal choice. Smaller values for w (which might occur for exams that cannot be failed without very serious repercussions) generally result in larger time investments, but this effect is much more evident for the no-resit condition than for the resit condition. Thus, the model predicts smaller values of w to be associated with stronger resit effects (see also [9]).

While we assumed no forgetting in the present study, this assumption would seem unrealistic and perhaps also unfortunate as forgetting intuitively might make the option of a resit a less attractive one, as it would involve having to spend extra study time to regain some of the knowledge acquired earlier in preparing for R1. This intuition is borne out by simulations of the model in which forgetting is activated. For instance, 50% forgetting of the knowledge gained earlier resulted in a marked reduction (by some 40%) of the resit effect, with w set at 0.1. These mitigating effects of forgetting on the resit effect were much less evident, however, at smaller values of w (in accordance with results from Kooreman [30], who reported no impact of forgetting on the resit effect for the very small values of w used in his model). The question of whether or not to include forgetting in the model is complicated further by the fact that it is not actual forgetting that is at issue here but students’ beliefs about their own forgetting. Available evidence points towards a strong stability bias with respect to learning and forgetting, i.e. students generally have rather unrealistic beliefs regarding the quality and stability of their learning (for review, see [31]). Empirical studies would seem required to determine the degree to which considerations of forgetting impact the way in which students deal with the opportunity of a resit.

Finally, in the present study participants were explicitly motivated to achieve a minimal passing grade with the least amount of study time. Surely, many students will have higher aspirations and, for various reasons, aim at passing an exam with flying colors. An important question therefore is whether such students might be immune to the siren call of a resit opportunity. Unfortunately, provided that study time is also a limited commodity for such students, the model predicts that while they will obviously invest substantially more study time in general, the resit effect will be just as apparent, and in absolute terms even larger, for them as compared to less ambitious students, when access to the resit is unconditional. Interestingly, the model also predicts that the resit effect for the more ambitious subcategory of students can be effectively eliminated if access to the resit is made conditional on obtaining a failing grade on R1 (in which case cutting back on study time for R1 would entail a very real risk of being stuck with a disappointingly low passing grade). This latter example also illustrates how a computational model of the way students deal with resit policies, when its core assumptions have been properly validated, may serve as an important instrument to inform policy decisions in higher education [32].

### Conclusions

Remarkably, even though resit exams are widely prevalent in higher education, little is known about how they might affect students’ study-behavior. In the current study, we presented and tested a mathematical model that can be used to derive predictions for how rational students would deal with the opportunity of a resit exam. By explicitly modeling knowledge accumulation as a function of study time, and by employing basic derivations of probabilistic outcomes on multiple-choice tests as a function of accumulated knowledge, our model can be said to offer a closer approximation of exam preparation in real life than the model proposed by Kooreman [9]. Evidently, however, there are many regards in which our model can be expanded further, for instance by incorporating students’ ambitions to obtain high grades instead of merely passing an exam, by incorporating assumptions about the depreciation of knowledge, and by incorporating assumptions about the temporal discounting of perceived costs of study-time investment for a resit. By demonstrating that the current, simple version of the model produces accurate predictions for study-time investment in a laboratory task, the current study can be said to mark the starting point of a line of inquiry that might contribute to the development of a proper scientific foundation for resit policies in higher education.
